# Developmental changes in using verbal self-cueing in task-switching situations: the impact of task practice and task-sequencing demands

**DOI:** 10.3389/fpsyg.2013.00940

**Published:** 2013-12-17

**Authors:** Jutta Kray, Hanna Gaspard, Julia Karbach, Agnès Blaye

**Affiliations:** ^1^Department of Psychology, Development of Language, Learning and Action, Saarland UniversitySaarbrücken, Germany; ^2^Center for Educational Science and Psychology, University of TübingenTübingen, Germany; ^3^Educational Psychology, Saarland UniversitySaarbrücken, Germany; ^4^Laboratoire de Psychologie Cognitive, UMR 7290, Aix-Marseille UniversitéMarseille, France

**Keywords:** task switching, verbal self-cueing, working memory, practice, childhood

## Abstract

In this study we examined whether developmental changes in using verbal self-cueing for task-goal maintenance are dependent on the amount of task practice and task-sequencing demands. To measure task-goal maintenance we applied a switching paradigm in which children either performed only task A or B in single-task blocks or switched between them on every second trial in mixed-task blocks. Task-goal maintenance was determined by comparing the performance between both blocks (mixing costs). The influence of verbal self-cueing was measured by instructing children to either name the next task aloud or not to verbalize during task preparation. Task-sequencing demands were varied between groups whereas one group received spatial task cues to support keeping track of the task sequence, while the other group did not. We also varied by the amount of prior practice in task switching while one group of participants practiced task switching first, before performing the task naming in addition, and the other group did it vice versa. Results of our study investigating younger (8–10 years) and older children (11–13 years) revealed no age differences in beneficial effects of verbal self-cueing. In line with previous findings, children showed reduced mixing costs under task-naming instructions and under conditions of low task-sequence demands (with the presence of spatial task cues). Our results also indicated that these benefits were only obtained for those groups of children that first received practice in task switching alone with no additional verbalization instruction. These findings suggest that internal task-cueing strategies can be efficiently used in children but only if they received prior practice in the underlying task so that demands on keeping and coordinating various instructions are reduced. Moreover, children benefitted from spatial task cues for better task-goal maintenance only if no verbal task-cueing strategy was introduced first.

## Introduction

In recent years, a growing number of studies have investigated the relationship between the use of language, such as verbal self-cueing strategies, and developmental changes in cognitive control [for reviews, see (Winsler et al., 2009; Cragg and Nation, 2010; Kray and Ferdinand, 2013)]. These studies found that younger children benefitted more from verbal self-cueing for maintaining task goals than older children and adults (Kray et al., 2008) and were less efficient in translating arbitrary cues into task goals than older children (Chevalier and Blaye, 2009; Blaye and Chevalier, 2011), suggesting that they probably do not spontaneously apply verbal strategies. The general goal of the present study was to further specify conditions in which such verbal strategies are beneficial for the regulation of task-switching behavior. In particular we were interested in examining whether developmental changes in benefitting from verbal self-cueing in a task-switching situation are dependent on different working-memory demands that were varied by the amount of practice and task-sequencing demands. On the one hand, demands on working memory were investigated as a function of prior practice in task execution and switching between them (henceforth termed task practice). Here we expected that prior task practice will lead to an automatization of the involved processes so that demands on keeping task instructions in mind in a task-switching situation will be reduced, making it easier to apply additional verbal instructions. On the other hand, we varied the demands on keeping track of task goals and their sequence during switching in order to examine whether beneficial effects of verbal labeling the next task goal become larger without the presence of external task cues.

### Developmental changes in task switching

To assess developmental changes in goal maintenance and cognitive flexibility in middle childhood, researchers have used several variants of the task-switching paradigm, such as cued task-switching paradigms in which the next task is indicated by a cue (e.g., Crone et al., 2004; Karbach and Kray, 2007; Manzi et al., 2011) or alternating runs paradigms in which the sequence of tasks is predictable and subjects have to switch the task on every second trial (e.g., Kray et al., 2008, 2010, 2012b; Karbach and Kray, 2009; for a review, see Cragg and Chevalier, 2012). In both of these types of paradigms, participants have to perform two easy categorization tasks, such as classifying objects as a dog or a car (in task A) or classifying their color as red or blue (in task B). The two tasks have either to be performed in separate blocks (single-task blocks) or subjects have to switch between both tasks A and B within the same block (mixed-task blocks). This design allows calculating different types of costs, reflecting different components of cognitive control (for a recent review, see Kiesel et al., 2010): Switching costs can be defined as difference in mean performance between switch trials (switching from task A to task B or from task B to task A) and repetition trials (repetition of task A or task B), and are assumed to reflect the ability to flexibly adapt to new task rules on a trial-to trial basis (e.g., Rogers and Monsell, 1995). Mixing costs are determined as difference in performance between mixed-task trials and single-task trials and are assumed to reflect the ability to maintain and select task goals (e.g., Kray and Lindenberger, 2000). Nearly all developmental studies found an increase of this ability during middle childhood as well as larger age-related changes in task-goal maintenance than in cognitive flexibility, suggesting the ability of switching between task and rules develops earlier (e.g., Cepeda et al., 2001; Crone et al., 2004; Kray et al., 2004, 2008, 2012a; Reimers and Maylor, 2005; Dibbets and Jolles, 2006; Karbach and Kray, 2007; Manzi et al., 2011). Improvements in cognitive flexibility during middle childhood are sometimes found (Huizinga and van der Molen, 2011), especially when irrelevant task attributes have to be ignored, that is, on incompatible trials (e.g., Cragg and Nation, 2009; Gupta et al., 2009). Considering this general pattern of findings, our intervention studies in recent years aimed at improving task-goal maintenance (Kray et al., 2008, 2010; Karbach and Kray, 2009; Karbach et al., 2010) and in the present study we focused our investigation on the usefulness of verbal self-cueing in middle childhood.

### Language and task switching

The claim that language has an important function for the regulation of behavior is not new in Psychology and has been promoted, among others, by Vygotsky and Luria (Luria, 1961; Vygotsky, 1962). However, only recently researchers have started to systematically investigate the role of verbal processes for efficiently maintaining task-set instructions as well as switching between them in adults (Baddeley et al., 2001; Emerson and Miyake, 2003; Miyake et al., 2004; Saeki and Saito, 2004; Bryck and Mayr, 2005) as well as in middle childhood (Kray et al., 2004, 2008; Karbach and Kray, 2007) and in older adults (Kray et al., 2008, 2010; Karbach et al., 2010). Most of these studies have found that mixing costs, but not switching costs, were larger when the use of inner speech was disrupted by articulatory suppression (e.g., Emerson and Miyake, 2003; Saeki and Saito, 2004). Hence, when subjects had to perform an additional verbal task (such as naming days of the week aloud) while switching between tasks, mixing costs were substantially higher. This increase was not obtained if subject had to perform a secondary motor task (such as a tapping task), suggesting that the increase in mixing costs was not simply due to an increase of dual-task demands but was instead specific to the disruption of verbal processes (e.g., Baddeley et al., 2001; Emerson and Miyake, 2003).

There is also evidence from a number of studies that the increase of mixing costs by articulatory suppression is even larger if other external (task) cues are either fully absent or if task cues are rather abstract or less transparent. This suggests that the impact of inner speech is largest when no other external task cues support task maintenance and retrieval or when abstract task cues needed to be translated into a semantic task-goal representation (e.g., Baddeley et al., 2001; Emerson and Miyake, 2003; Miyake et al., 2004; Chevalier and Blaye, 2009; Blaye and Chevalier, 2011). Finally, it has been shown that verbal suppression effects on mixing costs were larger under higher demands on working memory requiring participants to keep track of the task order as compared to conditions in which spatial cues supported the maintenance of the task sequence in mixed blocks. Therefore, some researchers have suggested that verbal processes have a specific task-sequencing function for the regulation of behavior (cf. Bryck and Mayr, 2005). In sum, it seems that the recruitment of inner speech processes is particularly needed in the absence of external cues in order to maintain relevant task goals or the sequence of tasks in switching situations (cf. Emerson and Miyake, 2003; Bryck and Mayr, 2005; Logan and Schneider, 2006).

Capitalizing on task-switching studies that have demonstrated the specific role of inner speech processes by applying articulatory suppression approaches, the particular interest of our recent studies was on examining the effects of verbal self-cueing (or self-instructions) on the regulation of behavior. In these studies, we investigated whether naming the next task goal under mixed-tasks conditions was helpful for maintaining task goals and switching between them. Therefore, we instructed participants to name the next task prior to stimulus presentation (Kray et al., 2004, 2008, 2009, 2010; Karbach and Kray, 2009; Karbach et al., 2010). To maximize the impact of verbal processes in this study, subjects received no task cues and had to keep track of the task sequence in mixed-task blocks. Results of these studies indeed indicated that mixing costs were largely reduced when subjects used verbal self-instructions during task switching (Kray et al., 2004, 2008, 2010), suggesting, in line with Luria (1961) and Vygotsky (1962), that verbal processes have an important self-cueing function for the regulation of task-switching behavior. For the purpose of the present study we will examine whether benefits of verbal labeling the next task on task switching will be reduced when demands on keeping track of the task sequence are reduced, that is, if external task cues can be used to facilitate the retrieval of the next task.

### Verbal self-cueing and developmental changes in task switching

From a developmental and applied perspective, an important question concerns the usefulness of verbal cueing as a tool for improving the regulation of behavior in age ranges that are known to have impairments in cognitive control functioning (cf. Kray and Ferdinand, 2013). During middle childhood, cognitive control is still maturing, and as noted before, mixing costs are reduced with increasing age throughout childhood development. Given the known beneficial effects of verbal cueing for efficiently switching between tasks, it was of theoretical as well as practical interest whether younger children are as able as adults to profit from verbal cueing strategies or whether they have deficits in applying such strategies. Results of our previous studies indicated that younger children between 7 and 9 years of age even benefitted more than older children (age range = 11–13 years) and younger adults, that is, the reduction of mixing costs was larger under task-naming conditions as compared with silent condition or a task-irrelevant verbalization condition (e.g., Kray et al., 2008). Hence, it seems that younger children use verbal self-cueing strategies for the regulation of behavior less spontaneously than older ones and young adults, but are able to use them if they are explicitly instructed to do so.

However, it should be noted that participants in our previous study already had some practice in task switching (one session) before receiving and performing the task-naming instruction (Kray et al., 2008). Hence, such practice in implementing task-set instructions and switching between them should reduce the working-memory load and facilitate memorizing and applying the additional verbalization task. Evidence for the effects of practice in task switching on verbal self-cueing benefits is rather scarce so far and is limited to studies on adults. In these studies, effects of verbal-labeling benefits were larger when subjects had already practiced the switching task without the verbal task or if they had intensive practice on both tasks (Karbach et al., 2010; Kray et al., 2010). Indirect evidence that a higher load on working memory is a critical factor for verbal benefits on task switching in childhood also comes from a task-switching training study. Here, we found that children between 8 and 10 years of age, but not adults, showed less transfer of task-switching training if new tasks and labeling instructions had to be remembered in every training session, suggesting that the higher load on working memory in this training condition (maintaining two new task instructions and verbalization instructions in each training session) hampered the transfer of switching training in children (Karbach and Kray, 2009). Hence, so far we do not know whether the reported evidence on the beneficial effects of verbal self-cueing in task-switching situations are limited to situation of prior task practice. Therefore, we investigated in the present study whether such beneficial effects are also obtained if children had not prior practice in the switching task and memory demands to remember the different instructions for the tasks, the switching, and the verbalization are quite high. Moreover, given that working-memory capacity is still more limited in middle childhood than in adolescence (Gathercole et al., 2004), the requirement to memorize not only the task rules but also the verbalization instructions may exceed younger children's working-memory capacity, therefore, limiting the usefulness of verbal self-cueing strategies especially in younger children even after prior practice in task switching.

Finally, children rely more strongly on environmental cues before the regulation of behavior becomes more goal-directed throughout childhood development (Cragg and Nation, 2010). As already noted, spatial cueing of the task sequence has been assumed to reduce working-memory load for keeping track of the task sequence, and is therefore, also expected to influence verbal self-cueing effects on task switching. If older children rely less on environmental cueing and more on internal cueing to guide behavior, we may expect larger age differences in verbal benefits on task switching when additional task cues are absent.

### Study goals

Recent studies showed that children can use verbal self-cueing instructions as a strategy to enhance the task-goal maintenance and selection, but often do not spontaneously apply such strategies (Kray et al., 2008; Chevalier and Blaye, 2009). The goal of the present study was to further specify conditions under which such verbal benefits occur throughout childhood development. In particular we aimed at determining whether age differences in verbalization benefits on switching tasks are influenced by demands on working memory. The impact of working memory was determined by varying the presence vs. absence of spatial task cues that support the maintenance of the task sequence in an alternating runs paradigm and the amount of learning experience in task switching.

To do this, we used an alternating runs paradigm and instructed participants to switch between two tasks A and B on every second trial. In task A (the picture task), participants were to decide whether a picture belonged to the category of dogs or cars and in task B (the color task) they were to decide whether the color of an object was blue or orange. To examine verbalization effects, children performed two conditions: they either verbalized the next task goal by saying “picture” or “color” prior to stimulus presentation (task-naming condition) or they did not verbalize the next task (silent control condition).

**Figure 1:**
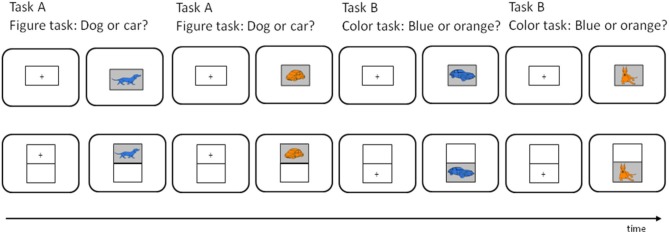
**Example of an AABB run in the switching task for the 1-grid group (upper trials) and the 2-grids group (lower trials)**. In the picture task (task A), children were to decide whether the stimulus was a dog or car, and in the color task (task B), they were to decide whether the stimulus was blue or orange. In the task-naming condition children were instructed to name aloud the upcoming task to the onset of the fixation cross. In both groups this would be “picture,” “picture,” “color,” “color” for the four trials, respectively.

For the specific purpose of this study, children were assigned to one of two groups: A group with high task-sequence load performed the task without any spatial task cues, i.e., demands on keeping track of the task sequence were high, because all stimuli appeared in one *grid* (see Figure 1). In contrast, the group with low task-sequence load performed the switching task with spatial task cues, that is, the stimuli appeared in one of two *grids* (cf. Bryck and Mayr, 2005; Kray et al., 2010). An upper grid was indicative of one task (task A: picture task) and a lower grid indicated that the other task (task B: color task) was to be performed (see Figure 1). Hence, demands on keeping track of the task sequence were low because the spatial position of stimulus appearance was a valid task cue. Furthermore, to assess the effects of previous experience (i.e., practice) in task switching, one group of children first performed the switching task in the silent control condition while the other group performed the task-naming condition first. Given that the ability to maintain task goals in switching situations as well as working memory increase until early adolescence, we compared task-switching performance and task-naming effects in 8–10 year-old children to 11–13 year-old children.

We expected to replicate previous findings, that is, reliable mixing costs (for a review, Kiesel et al., 2010) that should be reduced with increasing age (e.g., Cepeda et al., 2001; Crone et al., 2004; Dibbets and Jolles, 2006; Karbach and Kray, 2007; Kray et al., 2008, 2012a; Manzi et al., 2011). Mixing costs should also be reduced under conditions of spatial task-cueing with a lower task-sequence load, that is, mixing costs should be smaller in the 2-grids groups than in the 1-grid group (e.g., Bryck and Mayr, 2005). Similar to results obtained in adult samples, we also expected that benefits of verbal cueing on task switching should be larger if children already had already prior practice in task switching, (cf. Karbach et al., 2010; Kray et al., 2010). That means that the reduction of mixing costs under task-naming conditions compared to the silent condition is may only found for groups of children that first performed the switching task without verbalization. The verbal benefits on task switching may will be smaller for younger than for older children, even in the groups that received prior practice in task switching. Finally, we predicted that the reduction of mixing costs under task-naming conditions should be smaller in the 2-grids groups then in the 1-grid groups, as children rely less on verbal processes to keep track of the task sequence in the present of external cues. As younger children strongly rely on external cues to guide behavior, we also expected smaller age differences in verbalization effects on task switching in the 2-grids groups than in the 1-grid groups.

## Materials and methods

### Participants

Overall, 91 children participated in this study. They were recruited from a subject pool at Saarland University and were paid 7.50 € per hour. All children's parents provided informed consent. Two children were excluded from data analyses: One 8-year-old child was not motivated to perform the experimental task until the end, and one 11-year-old child showed extremely high reaction times (RT) (more than three standard deviations above the mean of the corresponding age group). The final sample consisted of 49 younger children (*age range* = 8–10 years) and 40 older children (*age range* = 11–13 years). Characteristics of the sample are summarized in Table 1.

**Table 1:** **Descriptive statistics for the participants**.

**Statistic**	**Age group**			
		**Younger children**		**Older children**	
	**1 grid**	**2 grids**	**1 grid**	**2 grids**
*N*	24	25	20	20
Males/females	18/6	16/9	10/10	10/10
Age range	8–9	8–10	11–13	11–13
Mean age (*SD*)	9.4 (0.5)	9.3 (0.6)	12.2 (0.6)	12.1 (0.5)
Speed of processing score	38.7 (7.4)	37.6 (6.0)	41.8 (6.8)	42.6 (6.6)
Working memory score	5.3 (1.8)	5.2 (1.5)	6.7 (1.8)	6.8 (1.6)

To control for between-group differences in intellectual variables (for a description, see section Apparatus, Stimuli, and Tasks) between the 1-grid and 2-grids groups, we run an analysis of variances (ANOVA) with the two between-subjects factors Age group (younger vs. older children) and Memory load (1-grid vs. 2-grids). As expected from the developmental literature (e.g., Gathercole et al., 2004; Li et al., 2004), we obtained reliable age differences in a test of speed of processing, *F*_(1, 85)_ = 7.89, *p* < 0.01, η^2^_*p*_ = 0.09, that is, older children (*M* = 42.2; *SD* = 6.6) reached a higher score than younger children (*M* = 38.1; *SD* = 6.7), as well as in a working-memory test, *F*_(1, 85)_ = 17.29, *p* < 0.001, η^2^_*p*_ = 0.17, again indicating that older children (*M* = 6.7; *SD* = 1.7) performed better than younger children (*M* = 5.2; *SD* = 1.6). Importantly, children in the 1-grid and 2-grids groups did not significantly differ in speed of processing (*p* = 0.95) and working-memory capacity (*p* = 0.95).

### Apparatus, stimuli, and tasks

Two psychometric tests were used to measure speed of processing and working-memory span. For speed of processing, we administered a color-naming test. Children saw a sheet with a template in the top row assigning four different colors to four different shapes (yellow circle, blue cross, red triangle, and green square). Below the template, uncolored shapes were shown and children were instructed to name the corresponding colors as quickly and accurately as possible. The test score was the number of correctly named colors after 45 s. The task in the working-memory span test was to repeat aloud a sequence of digits in the reversed order of presentation (adapted from Wechsler, 2003). The length of the sequences ranged from two to eight digits and the task included two items per range. The test score was the number of items that were correctly recalled.

For the task-switching experiment, we used IBM compatible laptops (Dell™ Latitude™ D820) for data collection. The stimuli were presented on a WXGA 15.4-inch color monitor on a white background. Responses were registered with the q- and the p-keys of the laptop keyboard that were equipped with two color-coded attachment pieces. The experiment was programmed with the software package E-Prime 1.1. Visual stimuli consisted of pictures of three different dogs and cars that were each presented in three different shades of orange and blue.

Participants were instructed to perform two different tasks, a “picture” task (task A) and a “color” task (task B). In the picture task, participants were asked to decide whether the picture showed a dog or a car. In the color task, they had to decide whether the picture was in orange or blue. In both tasks, subjects pressed the left (q-key) or the right (p-key) key with the left or the right index finger, respectively. In half of the blocks, participants were instructed to verbalize the task in addition, that is, they were asked to name the next task that had to be performed. In particular, they said “picture” or “color,” depending on the task, and started to verbalize with the onset of the fixation cross in each trial.

### Procedure

Children were tested in a one-session experiment. At the beginning, their parents completed a short demographic questionnaire and provided informed consent. Children started by performing two psychometric tests (the color-naming test and the working-memory span test; see Apparatus, stimuli, and tasks) followed by the switching task.

The task-switching experiment was divided into two parts: A verbalization condition, in which the switching task was performed under verbal self-instructions (i.e., naming the next task) and a silent condition without verbalization. Half of the participants first performed the silent condition and continued with the task-naming condition and vice versa. Each part was divided into an introduction phase and an experimental phase. The introduction phase consisted of two single-task blocks (one block of task A and one block of task B) followed by two mixed-task blocks. In the testing phase, participants performed four single-task and four mixed-task blocks. In mixed-task blocks, the task sequence was always AABBAABBAA… Two single-task blocks (task A and task B) and two mixed-task blocks were always grouped together. The procedure in the task-naming condition was identical, except that subjects were additionally instructed to verbalize to the onset of the fixation cross. In total, participants performed eight single-task and eight mixed-task blocks.

Each block consisted of 17 trials, while the first trial in each block was not analyzed, given that it was neither a switch nor a repetition trial. Both single- and mixed-task blocks consisted of an equal number of four stimulus types (orange dogs, blue dogs, orange cars, blue cars). In addition, mixed-task blocks consisted of an equal number of repetition and switch trials.

Experimental trials started with the presentation of a fixation cross for 1400 ms. Then, the stimulus appeared and was presented until a response was registered. To reduce individual differences in beginning with the onset of task preparation and task verbalization, the time interval between the response and the next fixation cross was only 25 ms. Before each experimental block, an instruction window appeared, indicating whether task A, task B, or both tasks had to be performed, and whether naming of the next task was required in addition. After each block, feedback about subject's mean response time and percentage of errors was provided. Subjects were instructed to respond as quickly and as accurately as possible. In addition, if children did not verbalize trials in the previous block, they were reminded by the instructor to do so. After completing the first half of the switching task, participants had a short break of 5–10 min.

### Design

The two experimental factors Block type (single vs. mixed) and Verbalization (task naming vs. silent) were manipulated within subjects. The other three factors were between-subjects factors: First, the sequence of verbalization (practice) was varied across subjects to examine practice effects, that is, one group of children received prior practice in task switching before doing the verbalization task in addition, while the other group did it vice versa. Second, task-sequence load (1-grid vs. 2-grids) was manipulated between subjects. In the high task-sequence load condition, all stimuli were presented in one grid at the center of the screen, so that the demand of keeping track of the task sequence was increased (see Figure 1 at the top). In the low task-sequence load condition, the stimuli were presented in one of two grids (upper or lower half of the screen) with the position serving as a cue: The upper position indicated that task A (the picture task) was required and the lower position that task B (the color task) had to be performed. The third between-subjects factor was Age group (younger vs. older children). In sum, we used a 2 (block type) × 2 (verbalization) × 2 (practice) × 2 (task-sequence load) × 2 (age group) design.

## Results

ANOVAs were performed for latencies and error rates in the switching task. For both analyses, we excluded nine blocks with seven or more errors in verbalization or task performance (one single-task block of one subject and eight mixed-task blocks of four different subjects). The analysis of error rates was focused on incorrect responses in the switching tasks as verbalization errors were mostly omissions in mixed blocks and generally rather low in the two age groups (3.7% for younger children and 1.4% for older children). For the analysis of latencies, incorrect responses as well as latencies faster than 200 ms and slower than 3000 ms were excluded (2.8% for the younger children and 0.4% for the older children). Except mentioned otherwise, all analyses were based on log-transformed RT because they are less sensitive to age differences in baseline conditions (cf. Kray and Lindenberger, 2000).

### Analysis of error rates

We first analyzed the error rates in the switching task. Error rates as well as mixing costs and verbalization effects are shown in Table 2. They were higher in mixed-task blocks than in single-task blocks for all four groups, and children made more errors under task naming than under silent conditions, especially when they had to switch between tasks. These observations were confirmed by an ANOVA including the within-subjects factors Block type and Verbalization and between-subjects factors Practice, Task-sequence load, and Age group.

**Table 2:** **Mean error rates (SE) as a function of block type (single-task/mixed-task), age group (younger children/older children), verbalization condition (silent control/verbalization), and practice (silent control condition first/verbalization condition first)**.

	**Silent control**	**Task naming**	**Verbalization effect**
**WITH PRACTICE IN TASK SWITCHING (SILENT CONTROL CONDITION FIRST)**			
**Younger children**			
Single blocks	4.88 (0.78)	5.48 (0.92)	0.60 (0.89)
Mixed blocks	8.58 (1.23)	10.29 (0.91)	1.71 (1.43)
Mixing costs	3.71 (1.18)	4.81 (0.75)	1.10 (1.40)
**Older children**			
Single blocks	3.20 (0.35)	5.08 (0.76)	1.88 (0.72)
Mixed blocks	5.36 (0.89)	9.77 (1.45)	4.40 (1.14)
Mixing costs	2.16 (0.86)	4.69 (1.16)	2.53 (0.95)
**WITHOUT PRACTICE IN TASK SWITCHING (VERBALIZATION CONDITION FIRST)**			
**Younger children**			
Single blocks	3.58 (0.64)	3.41 (0.61)	−0.17 (0.62)
Mixed blocks	7.55 (1.19)	9.35 (1.18)	1.80 (1.18)
Mixing costs	3.97 (1.20)	5.95 (1.06)	1.97 (1.07
**Older children**			
Single blocks	6.25 (0.94)	6.82 (1.12)	0.57 (1.14)
Mixed blocks	8.98 (1.29)	12.19 (1.21)	3.20 (1.32)
Mixing costs	2.73 (0.93)	5.36 (0.98)	2.63 (1.20)

*Mixing costs = mixed blocks − single blocks; Verbalization effect = task naming − silent control condition. Positive values of difference scores indicate costs and negative values benefits*.

Results indeed revealed a main effect of Block type, *F*_(1, 81)_ = 96.41, *p* < 0.001, η^2^_*p*_ = 0.54, that is, higher error rates in mixed-task blocks than in single-task blocks, hence reliable mixing costs. There was also a main effect of Verbalization, *F*_(1, 81)_ = 13.70, *p* < 0.001, η^2^_*p*_ = 0.15, that was further qualified by an interaction with Block type, *F*_(1, 81)_ = 11.59 *p* < 0.01, η^2^_*p*_ = 0.13, indicating that children made more errors in mixed-task blocks than in single task blocks under task-naming conditions than under silent conditions. Of most interest in this study was whether the task-sequence load had an impact on the accuracy of responding in younger and older children. The results indicated that neither Age group, nor Task-sequence load had an effect on the accuracy of responding and neither one of the factors interacted with Block type and Verbalization. We only found a significant interaction between Age group and Practice, *F*_(1, 81)_ = 7.58, *p* < 0.01, η^2^_*p*_ = 0.09. *Post-hoc* analyses revealed that verbalization order had no effect in the group of younger children (*p* = 0.17), but as can be seen in Table 2, in the group of older children, error rates were generally higher for the group starting with the task-naming condition than for the group starting with the silent condition, *F*_(1, 36)_ = 5.63, *p* < 0.05, η^2^_*p*_ = 0.82.

All in all, error rates were rather low in all groups of children but increased when children had to switch between two tasks and even more under task-naming conditions, which has also been reported in previous studies (e.g., Kray et al., 2008, 2010). However, the reduction of task-sequence load by additional spatial cueing of the currently to-be-performed task (2-grids version) instead of keeping track of the task sequence (1-grid version) had no impact on the accuracy of responding or on task switching. For older children, the verbalization order had an effect on the accuracy of responding: The group that had to memorize and coordinate task switching with task naming instructions without prior practice (i.e., the group starting with the verbalization condition) produced a higher error rate, but there was no interaction with demands on cognitive control (i.e., block type). However, given that the error rates were relatively low, the impact of main experimental manipulations becomes more evident when analyzing latencies.

### Analysis of latencies

We first run an overall ANOVA for latencies including the same factors as for error rates to identify higher-order interactions with the factors Practice and Task-sequence load. In contrast to the results on error rates, we indeed found that mixing costs (Block type) interacted with Task-sequence load, *F*_(1, 81)_ = 4.66, *p* < 0.05, η^2^_*p*_ = 0.05, and Verbalization, *F*_(1, 81)_ = 5.95, *p* < 0.05, η^2^_*p*_ = 0.07, and the later effect was further qualified by practice effects: Block type × Verbalization × Practice: *F*_(1, 81)_ = 9.12, *p* < 0.01, η^2^_*p*_ = 0.10. Finally, we obtained an interaction between Verbalization and Practice, *F*_(1, 81)_ = 4.82, *p* < 0.05, η^2^_*p*_ = 0.06. To better understand the nature of these interactions, we conducted separate ANOVAs for the two verbalization-order conditions (see sections Benefits of Task Naming on Task Switching after Practice in Task Switching and Benefits of Task Naming on Task Switching without Practice in Task Switching).

#### Benefits of task naming on task switching after practice in task switching

**Figure 2:**
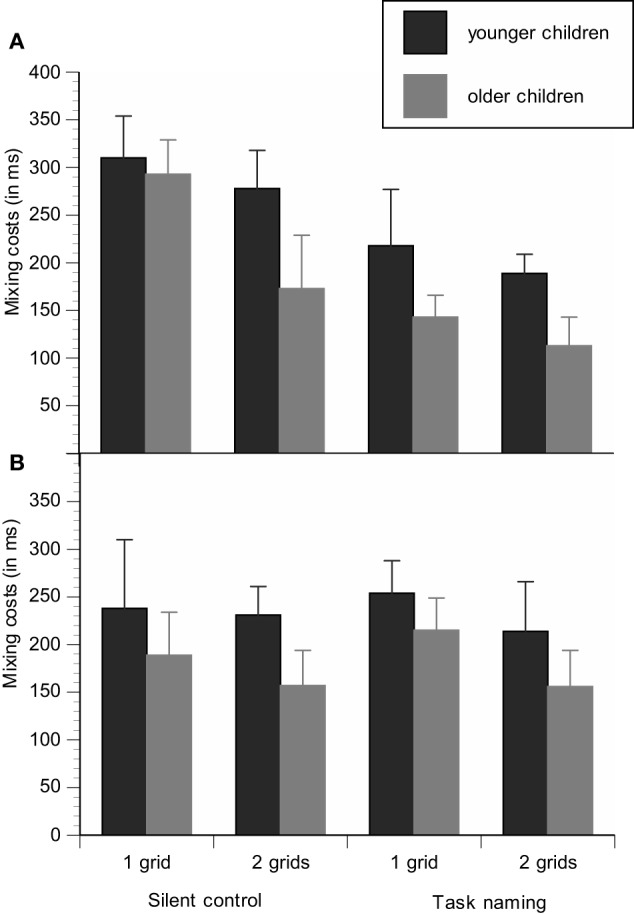
**Mixing costs are displayed as a function of age group (younger children/older children), task-sequence load (1 grid/2 grids), and verbalization condition (silent control/task naming) for the group with practice in task switching (silent condition first) (A)** and the group without practice in task switching (verbalization condition first) **(B)**.

**Figure 3:**
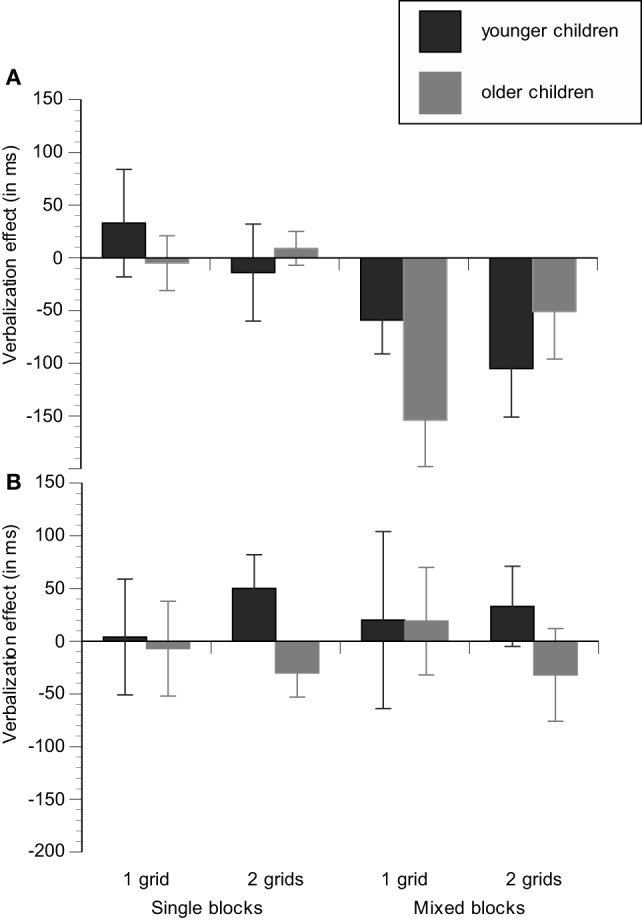
**Verbalization effects are displayed as a function of age group (younger children/older children), task-sequence load (1 grid/2 grids), and block type (single-task/mixed-task) for the group with practice in task switching (silent condition first) (A)** and the group without practice in task switching (verbalization condition first) **(B)**.

For the group that first received practice in task switching (i.e., first performed the silent control condition), we run an ANOVA including the between-subjects factors Age group and Task-sequence load and the within-subjects factors Block type and Verbalization. Latencies of all experimental conditions are reported in Table 3. Mixing costs are displayed as a function of age group (younger/older), task-sequence load group (1-grid/2-grids), and verbalization condition (silent/task naming) in Figure 2A and verbalization effects are shown as a function of age group (younger/older), task-sequence load (1-grid/2-grids), and block type (single blocks/mixed blocks) in Figure 3A.

**Table 3:** **Mean (SE) latencies (ms) as a function of block type (single-task/mixed-task), age group (younger children/older children), task-sequence load (1 grid/2 grids), and verbalization condition (silent control/verbalization) for the group with practice in task switching (silent control condition first)**.

	**Silent control**	**Task naming**	**Verbalization effect**
**HIGH TASK-SEQUENCE LOAD (1 GRID)**			
**Younger children**			
Single blocks	897 (88)	931 (63)	33 (51)
Mixed blocks	1207 (112)	1148 (106)	−59 (32)
Mixing costs	310 (44)	218 (59)	−92 (46)
**Older children**			
Single blocks	571 (26)	567 (32)	−5 (26)
Mixed blocks	864 (54)	710 (44)	−154 (44)
Mixing costs	293 (36)	143 (23)	−150 (33)
**LOW TASK-SEQUENCE LOAD (2 GRIDS)**			
**Younger children**			
Single blocks	853 (42)	839 (66)	−14 (46)
Mixed blocks	1133 (64)	1027 (71)	−105 (46)
Mixing costs	280 (40)	189 (20)	−91 (35)
**Older children**			
Single blocks	575 (35)	584 (27)	9 (16)
Mixed blocks	748 (84)	697 (50)	−51 (45)
Mixing costs	173 (56)	113 (30)	−60 (32)

*Mixing costs = RT mixed blocks − RT single blocks; Verbalization effect = RT task naming − RT silent control condition. Positive values of difference scores indicate costs and negative values benefits*.

The ANOVA revealed reliable age differences, *F*_(1, 41)_ = 31.44, *p* < 0.001, η^2^_*p*_ = 0.43, that is, slower latencies for younger children than for older children (see Table 3). As can also be seen in Table 3, for all groups latencies were slower in mixed-task blocks than in single-task blocks, indicating reliable mixing costs, *F*_(1, 41)_ = 184.18, *p* < 0.001, η^2^_*p*_ = 0.82.

In Figure 2A it seems that older children show smaller mixing costs than younger children. However, age differences in mixing costs were marginally significant on the basis of means, *F*_(1, 41)_ = 3.50, *p* = 0.07, η^2^_*p*_ = 0.08, but not significant for log-transformed RT (*p* = 0.75). However, for younger and older children mixing costs were reduced in the 2-grids group as compared with the 1-grid group, *F*_(1, 41)_ = 4.44, *p* < 0.05, η^2^_*p*_ = 0.10, suggesting better switching performance under conditions with lower task-sequence load. Mixing costs were also reduced under task-naming conditions as compared to the silent conditions for younger and older children, *F*_(1, 41)_ = 29.41, *p* < 0.001, η^2^_*p*_ = 0.42. Moreover, this effect of reduced mixing costs under task-naming conditions was more pronounced in the high task-sequence load group than in the low task-sequence load group, Block type × Verbalization × Memory load, *F*_(1, 41)_ = 5.25, *p* < 0.05, η^2^_*p*_ = 0.11, in line with the prediction that task naming results in smaller mixing costs especially under conditions in which external task cues that support task maintenance are missing.

Regarding verbalization effects, we found a significant main effect of Verbalization, *F*_(1, 41)_ = 7.21, *p* < 0.05, η^2^_*p*_ = 0.15. Verbalization effects are better seen in Figure 3A that shows that benefits of task naming were restricted to mixing blocks [*F*_(1, 42)_ = 23.14, *p* < 0.001, η^2^_*p*_ = 0.36] and were not present in single blocks (*p* = 0.85). Finally, the ANOVA revealed an interaction between Verbalization, Age group, and Task-sequence load, *F*_(1, 41)_ = 4.31, *p* < 0.05, η^2^_*p*_ = 0.10. Separate analyses for each age group indicated only a marginally significant interaction between Verbalization and Task-sequence load group for the older children (*p* = 0.10), but not for the younger children (*p* = 0.21), indicating that verbalization benefits on task performance were larger in tendency in the 1-grid condition.

#### Benefits of task naming on task switching without practice in task switching

For the group that first performed the verbalization condition, we run the identical set of analyses. Latencies of all experimental conditions are reported in Table 4. Mixing costs are displayed as a function of age group (younger/older), memory load group (1-grid/2-grids), and verbalization condition (silent/task naming) in Figure 2B and verbalization benefits are shown as a function of age group (younger/older), task-sequence load (1-grid/2-grids), and block type (single blocks/mixed blocks) in Figure 3B.

**Table 4:** **Mean (SE) latencies (ms) as a function of block type (single-task/mixed-task), age group (younger children/older children), task-sequence load (1 grid/2 grids), and verbalization condition (silent control/verbalization for the group without practice in task switching (verbalization condition first)**.

	**Silent control**	**Task naming**	**Verbalization effect**
**HIGH TASK-SEQUENCE LOAD (1 GRID)**			
**Younger children**			
Single blocks	898 (50)	902 (52)	4 (55)
Mixed blocks	1136 (84)	1156 (55)	20 (84)
Mixing costs	238 (72)	254 (72)	16 (72)
**Older children**			
Single blocks	600 (45)	593 (50)	−7 (45)
Mixed blocks	789 (62)	808 (52)	19 (51)
Mixing costs	189 (45)	215 (34)	26 (66)
**LOW TASK-SEQUENCE LOAD (2 GRIDS)**			
**Younger children**			
Single blocks	790 (53)	840 (56)	50 (32)
Mixed blocks	1021 (65)	1054 (81)	33 (38)
Mixing costs	231 (30)	214 (52)	−17 (31)
**Older children**			
Single blocks	551 (40)	521 (33)	−30 (23)
Mixed blocks	709 (69)	677 (63)	−32 (44)
Mixing costs	157 (37)	156 (38)	−1 (44)

*Mixing costs = RT mixed blocks − RT single blocks; Verbalization effect = RT task naming − RT silent control condition. Positive values of difference scores indicate costs and negative values benefits*.

The results only revealed reliable age differences, *F*_(1, 40)_ = 39.77, *p* < 0.001, η^2^_*p*_ = 0.81, and mixing costs, *F*_(1, 40)_ = 165.58, *p* < 0.001, η^2^_*p*_ = 0.50. Again, Figure 3B shows that mixing costs were lower in the group of older children than in the group of younger children, but reliable age effects on mixing costs were neither obtained on the basis of means, (*p* = 0.14), nor for log-transformed RT (*p* = 0.68). Mixing costs did not vary as a function of task-sequence load (see Figures 2B and 3B) and we neither found verbalization benefits (*p* = 0.57) nor interactions with any variable of interest. Hence, verbalization benefits on task switching strongly depend on prior practice of the switching task.

## Discussion

In this study, we investigated whether developmental changes in the use of verbal self-cueing (i.e., task naming) for efficiently switching between cognitive tasks are influenced by different demands on working memory. To vary memory demands on keeping track of the task sequence, one group of children performed an alternating switching task without external memory aid, and hence, demands on keeping track of the task sequence of AABB under mixed-task conditions were high, and the other group could use spatial task cues as external memory aid. It has been suggested that verbal processes are especially required and useful if other task cues supporting the maintenance of the task sequence are missing (e.g., Bryck and Mayr, 2005). In the other group, the order of tasks was also predictable, but the spatial position of the stimulus additionally indicated which one of the two tasks had to be performed in a given trial. Thus, we assumed that the working-memory demands in terms of maintaining the task sequence should be reduced and task naming would be less beneficial under these conditions. Another factor investigated in this study that is assumed to influence working-memory demands is the amount of prior practice in task switching, that is, whether children did the verbalization task with or without previous silent practice in task switching. Capitalizing on previous findings in adult samples (Karbach et al., 2010; Kray et al., 2010), we expected that task naming may only be beneficial if children had at least some practice in performing the two tasks and switching between them. Otherwise, working-memory demands are relatively high, as the task-naming instruction has to be maintained and coordinated on top of the two task-set instructions.

First, we replicated a number of findings that have already been reported in the literature. Our results indicated reliable mixing costs, that is, subjects were slower and made more errors in mixed-task blocks than in single tasks blocks, as shown in nearly all task-switching studies (cf. Kiesel et al., 2010). We also found partly that mixing costs were smaller in older children than in younger children, at least when costs were measured on the level of mean latencies (cf. Cepeda et al., 2001; Crone et al., 2004; Reimers and Maylor, 2005; Dibbets and Jolles, 2006; Karbach and Kray, 2007; Kray et al., 2008, 2012a; Manzi et al., 2011). However, taking the substantial baseline differences in latencies between both groups of children into account, the younger children in our study did not show proportionally larger mixing costs. Moreover, mixing costs on the level of error rates also did not significantly differ between younger and older children. One explanation for lacking age differences in mixing costs in the present study is that the younger children in the present study were somewhat older than in most other studies (e.g., Dibbets and Jolles, 2006; Karbach and Kray, 2007). However, we also intended to investigate even younger children (5–6 years old) in this study, but these children turned out to produce too many errors in this paradigm to obtain reliable findings from this age group.

The specific interest in the present study was whether children used verbal cueing to facilitate task-goal maintenance as a function of task practice and the presence of spatial task cues. At first our results clearly indicated that the usefulness of applying the task-naming strategy varied as a function of prior practice in task switching, similar to results found in adult samples (Karbach et al., 2010; Kray et al., 2010). Only the children who first practiced task switching before additionally verbalizing the task goals showed verbalization benefits in mixing blocks. In contrast to these findings, we did not obtain verbalization benefits or age differences therein if subjects had no prior practice in task switching. However, one should keep in mind that both the instructions for verbalizing on time (to the onset of the fixation cross) and for the two switching tasks and their corresponding response alternatives had to be memorized and coordinated for optimal task performance; it therefore, may not surprise that the optimal integration of these rules and their application needs some practice. Evidence from studies with older adults indeed showed that providing more practice for this integration process finally results in verbal labeling benefits (Karbach et al., 2010; Kray et al., 2010).

There is also evidence that the task-sequence load manipulation had an effect on task switching, but interestingly again only for the groups of children that practiced task switching first without verbal labeling. In line with our predictions we obtained smaller mixing costs in the groups with the 2-grids version than in the groups with the 1-grid version, suggesting that children made use of spatial task cues in order to facilitate the maintenance of the task sequence, thereby resulting in faster task switching in both age groups. In contrasts, for the groups that were introduced to the verbal labeling instruction from the beginning on, there was no difference whether additional spatial cues were presented or not. Hence, if children were already instructed to an efficient verbal cueing strategy, they did not further improve the maintenance of tasks by receiving additional spatial task cues. However, this finding was restricted to the analysis of latencies, as the reduction of task-sequence load did not support the accuracy of task switching, perhaps because error mixing costs were generally low in both age groups.

Age differences (at least in tendency) in verbal benefits as a function of the task cueing were only found for general speed of responding and were not specific to the maintenance of task goals, in contrast to our predictions. For younger children, verbalization benefits did not significantly differ between the conditions with or without the presence of task cues, although they showed a pattern in opposite to the older children, suggesting that they used verbal strategies relatively independent of the task context. In contrast, older children adapted their verbal strategy to the demands of the situation: Verbal benefits were larger in the 1-grid group, suggesting that older children more strongly relied on verbal strategies in the absence of spatial cues than in the presence of such cues (see Figure 3B). However, given the general lack of substantial age differences in the present study and that these interactions were only significant in tendency they should interpreted with caution and replications with age groups varying in a broader age ranges are indicated for future research.

In recent years, a number of researchers addressed the role of inner speech for the development of cognitive control (for reviews, see Winsler et al., 2009; Cragg and Nation, 2010; Kray and Ferdinand, 2013). Inner speech is assumed to have an important function for (1) selecting and activating relevant task sets, especially if task cues are abstract or not transparent (Baddeley et al., 2001; Emerson and Miyake, 2003; Miyake et al., 2004; Logan and Schneider, 2006; Chevalier and Blaye, 2009; Blaye and Chevalier, 2011) and (2) sequencing and memorizing the task sequence if the order of tasks is predictable and task cues are missing (cf. Bryck and Mayr, 2005). We recently suggested that verbal labeling supports these functions by optimizing task preparation (Kray et al., 2010). Hence, the instruction to apply verbal labeling on each trial supports children's constant task engagement and the additional instruction to verbalize “to the onset of the fixation cross” tells them exactly “when” to prepare. Results of this study are consistent with these theoretical considerations but clearly show that the beneficial function of verbal labeling is limited to occasions with previous practice in the cognitive control tasks. Thus, verbal labeling only facilitates the selection and activation of task goals, if the task representation (task goals and their response alternatives) has already been well established. How much practice younger and older children need to form this kind of representation is beyond the scope of the present study and a matter for future research.

Furthermore, the results of the present study provide no answer to whether other strategies instead of verbal cueing are also beneficial and can support task preparation in middle childhood. From developmental studies in early childhood we know that pointing strategies can be as useful as verbal strategies for the regulation of behavior (e.g., Müller et al., 2004). Given that we had no further non-verbal conditions in our research design we cannot conclude that language processes such as verbal self-cueing is the best or only way to subserve the regulation the behavior.

In sum, results of the present study indicated that both younger and older children had smaller mixing costs under task-naming conditions, especially when external task cues were missing, after receiving prior practice in task switching. Hence, the control of task-switching behavior in middle childhood can be supported by verbal self-cueing but such beneficial effects of task naming primarily occurs if children had already practice in being in a switching situation, as demands on holding and coordinating several instructions in mind are reduced. Our findings also revealed that younger and older children had smaller mixing costs under lower task-sequencing demands, that is, when spatial task cues facilitated task maintenance. However, children made use of such spatial task cues primarily in situations in which verbal strategies were not instructed first. Given that verbal self-instructions can generally be seen as a useful tool for enhancing the control of behavior, at least one important question for future research is to determine how much practice in a given task is needed before verbal strategies can be beneficially applied at various ages across childhood development.

## Author contribution

Hanna Gaspard and Jutta Kray analyzed the data of the present study; Jutta Kray, Julia Karbach, and Agnès Blaye designed the study, and all authors contributed to the writing of the paper.

### Conflict of interest statement

The authors declare that the research was conducted in the absence of any commercial or financial relationships that could be construed as a potential conflict of interest.
